# Factors affecting primary care practitioners’ alcohol-related discussions with older adults: a qualitative study

**DOI:** 10.3399/BJGP.2020.1118

**Published:** 2021-08-24

**Authors:** Bethany Kate Bareham, Jemma Stewart, Eileen Kaner, Barbara Hanratty

**Affiliations:** Newcastle University Population Health Sciences Institute, Newcastle University, Newcastle upon Tyne.; Newcastle University Faculty of Medical Sciences, Newcastle University, Newcastle upon Tyne.; Newcastle University Population Health Sciences Institute, Newcastle University, Newcastle upon Tyne.; Newcastle University Population Health Sciences Institute, Newcastle University, Newcastle upon Tyne.

**Keywords:** ageing, attitude of health personnel, drinking behaviour, harm reduction, primary health care, qualitative research

## Abstract

**Background:**

Risk of harm from drinking is heightened in later life, owing to age-related sensitivities to alcohol. Primary care services have a key role in supporting older people (aged ≥50 years) to make healthier decisions about alcohol.

**Aim:**

To examine primary care practitioners’ perceptions of factors that promote and challenge their work to support older people in alcohol risk-reduction.

**Design and setting:**

Qualitative study consisting of semi-structured interviews and focus groups with primary care practitioners in Northern England.

**Method:**

Thirty-five practitioners (GPs, practice/district nurses, pharmacists, dentists, social care practitioners, and domiciliary carers) participated in eight interviews and five focus groups. Data were analysed thematically, applying principles of constant comparison.

**Results:**

Practitioners highlighted particular sensitivities to discussing alcohol among older people, and reservations about older people’s resistance to making changes in old age; given that drinking practices could be established, and promote socialisation and emotional wellbeing in later life. Age-related health issues increased older people’s contact with practitioners, but management of older people’s long-term conditions was prioritised over discussion of alcohol. Dedicated time to address alcohol in routine consultations with older people and training in alcohol intervention facilitated practitioners, particularly pharmacists and practice nurses.

**Conclusion:**

There are clear opportunities to support older people in primary care to make healthier decisions about alcohol. Dedicated time to address alcohol, training in identification of alcohol-related risks (particularly those associated with old age), and tailored interventions for older people, feasible to implement in practice settings, would help primary care practitioners to address older people’s alcohol use.

## INTRODUCTION

Alcohol use is a leading modifiable cause of disease and premature death.^1^ Older people (aged ≥50 years) are more likely to experience alcohol-related harm than people of any other age group.^2^^,^^3^ Physiological tolerance of alcohol decreases with age.^4^^,^^5^ Older adults often have long-term conditions that may be exacerbated by drinking, or take medications that interact harmfully with alcohol.^6^^,^^7^ Most older people living in higher-income countries use alcohol. Up to 45% of older drinkers consume alcohol at hazardous levels, where their intake puts them at risk of physical, psychological, or social harm.^8^

Primary care services have a key role in supporting older people’s decisions about alcohol use, raising awareness of the potential impact on their health.^9^^–^^16^ Promoting healthier lifestyles and preventing disease are encompassed in primary care.^17^ Screening and brief intervention to address hazardous alcohol use are incorporated in many primary care services.^14^^,^^16^^,^^18^ Older people view primary care practitioners as a key source of support for any concerns about their alcohol use,^15^^,^^18^ and expect that practitioners would identify any risks associated with their drinking.^15^ Many older people reduce their alcohol intake following intervention from practitioners.^19^

Most older adults in the UK who are at risk of harm from their drinking report that their primary care practitioners have never expressed concern about their intake.^10^ Existing qualitative evidence suggests practice to address alcohol use in health and social care for older adults is hindered by a number of barriers, which mean older people’s needs for support are unmet.^14^

Understanding the potential facilitators and barriers for addressing hazardous alcohol use among older adults within primary care is key to informing how practitioners can be supported to fulfil their role and meet older people’s support needs. This qualitative study drew on primary care practitioners’ perspectives to explore:

- opportunities and assets in primary care to support older people to make healthier decisions for their alcohol use;
- factors that facilitate primary care practitioners’ work to support older people’s decisions about alcohol use; and
- challenges that affect primary care practitioners’ work to support older people’s decisions for alcohol use.

## METHOD

### Sampling and recruitment

Practitioner groups with roles in addressing older people’s alcohol use were identified from previous literature^14^ and input from the advisory group of 10 older members of the public who use alcohol (further details published elsewhere^18^), who listed practitioner groups that had discussed alcohol use during care provision. Practitioners working in North East/North Cumbria, England, were recruited through invitations circulated via the Clinical Research Network, and flyers distributed on Facebook and Twitter. Purposive sampling ensured that individuals were included from a range of identified professions including GPs, district and practice nurses, healthcare assistants, pharmacists, dentists, social care practitioners, and domiciliary carers.

**Table:** How this fits in

Older people have heightened risk of alcohol-related harm, owing to age-related sensitivities to alcohol. Primary care practitioners can play an important part in supporting older people’s decisions about how they use alcohol; however, older people’s needs for support are often not met by their practitioners. This study suggests that dedicated time to address alcohol during routine consultations to manage older people’s health (for example, during long-term condition reviews, medicine use reviews, and health checks) provides clear opportunity to support older people’s decisions. However, management of older people’s health conditions was prioritised over discussion of alcohol in time-constrained consultations, and reservations about older people’s particular sensitivity to alcohol-related discussion, as well as resistance to making changes to their established drinking practices, could deter alcohol discussion with older adults. Practitioners with defined roles and dedicated time to address alcohol with older people, training on low-risk alcohol use, and tailored intervention specific to older patients may all help primary care staff support older people’s decisions.

### Data collection

Qualitative data were collected in 2017 using in-depth semi-structured interviews and focus groups. Interviews were face-to-face, or by telephone if preferred. These were audio-recorded and transcribed verbatim. Topic guides structured discussion around:

- practitioners’ experiences of addressing alcohol use in their work with older people;
- factors affecting whether and how practitioners supported older people with decisions about their drinking; and
- practitioners’ views about alcohol and older people’s alcohol use.

Practitioners were asked to focus on their experiences of discussing non-dependent drinking with patients aged ≥50 years. Brief interventions typically supplied within primary care are unlikely to be enough to address alcohol dependence; specialist support is more appropriate in such instances.^20^ Guides were amended across data collection to examine emerging issues. Practitioners were asked to respond to written vignettes, describing plausible scenarios of hypothetical older adults’ drinking practices, health state, and social circumstances. These encouraged practitioners to share their views on when an older person might benefit from their support for decisions about alcohol use. Data collection ceased at the point of theoretical sufficiency, where new data added little insight to arising issues.^21^

Characteristics of practitioners were recorded, capturing factors that influence practitioner views about alcohol use (listed in Table 1, as indicated in wider literature^14^^,^^22^^–^^24^).

**Table 1.** Sample characteristics (*N* = 35)

**Sample size**	***n***
**Sex, female**	25

**Provider occupation**	
GP	7
Practice/district nurse	6
Healthcare assistant	3
Pharmacist	2
Dentist	10
Social care practitioner	5
Domiciliary carer	2

**Age, years**	
20–29	11
30–39	9
40–49	9
50–59	5
≥60	1

**Participation**	
One-to-one interview^a^	8
Focus group 1: General practice team	10
Focus group 2: Social care practitioner team	5
Focus group 3: Dentist team A	3
Focus group 4: Dentist team B	7
Dyad: Domiciliary care team	2

**Years in practice^b^**	
0–10	16
11–20	10
21–30	6
≥31	2

**Ethnicity**	
White British	32
Black British	2
Black African	1

**Practice area (urban or rural)**	
Urban	16
Rural	19

a *GPs, practice and district nurses, healthcare assistants, and pharmacists participated in one-toone interviews.*

b *Missing,* n *= 1.*

### Data analysis

Qualitative analysis was guided by Braun and Clarke’s thematic analysis^25^ (as detailed elsewhere^15^). Constant comparisons and negative case analysis deepened understanding.^26^ An inductive approach was taken. To aid interpretation, emerging ideas were discussed with other researchers, and relevant theoretical literature was explored. The COM-B model^27^ informed identification of factors that may affect practitioners’ work to support older people’s decisions about alcohol use. This framework explains behaviour (supporting older people’s decisions about alcohol use) in terms of three elements:

- the individuals’ capabilities (practitioners’ psychological and physical capacity to provide support);
- opportunities (factors external to the practitioner that prompt or enable provision of support); and
- motivation (subconscious habits and conscious intention).

## RESULTS

Thirty-five primary care practitioners participated in eight interviews and five focus groups (2–10 participants, each made up of in-practice colleagues). Interviews lasted an average of 42 min, and focus groups an average of 38 min. Participant characteristics and focus group compositions are detailed in Table 1. A majority of practitioners were aged <40 years, female (*n* = 25), and white British. They had been in practice for 3–40 years, and most drank alcohol themselves. Details of practitioners’ work with older people and alcohol are provided in Supplementary Box S1.

Factors affecting practitioners’ work, reported in the following themes, have been categorised into elements of the COM-B model^27^ in Box 1.

**Box 1.** Factors affecting practitioners’ work to support older people to make healthier decisions in their alcohol use identified in participant narratives and described in reported themes; categorised into COM-B components^27^

**COM-B component**	**Factors, including theme where issue is described**
**Physical capability** (the physical strength, skills, and stamina to perform the behaviour)	n/a

**Psychological capability** (the knowledge and psychological skills, strength, and stamina to perform the behaviour)	Knowledge of general health recommendations, risks, and guidelines for alcohol use (Theme 3)
	Awareness of prevalence of hazardous alcohol use among older population (Theme 1)
	Knowledge of specific risks of drinking in old age (Theme 3)
	Knowledge of potential positive contributions of alcohol use to older people’s wellbeing (Theme 1)
	Interpersonal skills to raise potentially sensitive topic of alcohol with older people, and ask about intake (Themes 1 & 3)
	Intervention skills to give older people advice about their alcohol use, and motivate healthier decisions (Theme 3)

**Physical opportunity** (what the environment allows or facilitates in terms of time, triggers, resources, locations, and physical barriers, for example)	(Dedicated) time to raise the topic of alcohol use (Themes 2 & 3)
	Available alcohol use risk screening resources (Theme 2)
	Reminders, cues, and awareness campaigns to raise alcohol use with older people (Theme 2)
	Clear signposting options for additional support with alcohol (Theme 2)

**Social opportunity** (a social environment conducive for the behaviour to occur, that is, socially acceptable; encompassing interpersonal influences, social cues, and cultural norms)	Perceived sensitivities associated with discussing alcohol use, where ‘problematic’ use is stigmatised, but use is a cultural norm and viewed to be the older person’s prerogative (Theme 1)
	Rapport with patients increases acceptance of alcohol-related discussion (Theme 1)
	Alcohol-related discussion as a clear component of standard care increases acceptability of alcohol-related discussion (Theme 2)
	Links between alcohol use and care concerns (Theme 2)

**Reflective motivation** (self-conscious planning and evaluations to perform the behaviour; evaluations about the behaviour, that is, beliefs about what is good or bad)	Perception of specific professional roles for addressing older people’s alcohol use — where alcohol use was prioritised above other care tasks (Theme 3)
	Belief in capability to discuss alcohol use with older people, and provide intervention where appropriate (Theme 3)
	Expectations about older people’s receptivity to discussion and intervention (Theme 1)

**Automatic motivation** (processes such as wants and needs, desires, impulses, and reflex responses)	Empathy for the older person’s motivations to use alcohol, for example, loneliness and coping with stresses (Theme 1)

*n/a = not applicable. Theme 1 = Perceptions about how receptive older people are to alcohol-related intervention. Theme 2 = Processes and practicalities of addressing alcohol use. Theme 3 = Professional remit and addressing older people’s alcohol use.*

### Themes

#### Perceptions about how receptive older people are to alcohol-related intervention

Practitioners involved in alcohol screening and intervention (detailed in Supplementary Box S1) discussed the challenges of older people recognising when their drinking may represent a risk. Older people’s cultural view of ‘normal drinking’, which often involved daily alcohol use, could overlap with clinical views of hazardous use, particularly among the younger-old (‘baby-boomer’ generation):

*‘People do have, say, a glass or two of wine with their meal at night, and it seems to be a bit more socially acceptable. Getting people to look at it differently, that’s not very much on a daily basis, but actually, when you add it up and when you’re looking at how many units you’re having over a week, it maybe is a little bit too much. But it is sometimes difficult to get people to think like that.’*

(Nurse)

Practitioners who worked with people of different ages expressed views that older drinkers tended to consume little alcohol relative to younger ‘binge’ drinkers. They described instances where they had not considered alcohol as a cause of potentially related health issues with older adults, particularly those perceived to be low-level drinkers, such as the oldest-old and people in care homes:

*‘I did a visit to a care home a few weeks ago and did a liver function test on a patient, not looking for an alcohol problem. They were a bit abnormal …* [I didn’t ask about her drinking] *because she’s in a nursing home. It didn’t occur to me that in a nursing home somebody might be drinking to excess and that this might be a problem; it made me think I need to be more aware of that.’*

(GP)

Practitioners perceived drinking practices to be well established by old age. Many had experience of older people’s resistance to advice, and had low expectations for the success of any intervention to address alcohol use:

GP3: *‘The thing with the elders they’re* [saying] *“I’m eighty-five years old, I’ve been drinking a half bottle of whisky every day.” They’re less likely to change.’*

GP4: *‘It’s hard to motivate them.’*

GP5: *‘It’s difficult to argue against.’* (General practice focus group [GPFG])

However, older people’s concerns about the vulnerability of their health in old age was recognised as a motivating factor for healthier lifestyle choices:

*‘You certainly get people, as they’re getting older, coming in with more general concerns about their general health and what they can do to help themselves.’*

(GP)

Practitioners were all wary of discussing alcohol use, and felt that it could be a sensitive topic for older people. Implying that someone’s drinking may be ‘problematic’ raised issues of stigma. This meant that some avoided raising their concerns with older people, owing to apprehensions about how rapport may be affected were offence caused:

*‘I don’t usually interfere unless it’s a problem. And even then it will fracture your relationship, of course it will and it’s really difficult to get them engaged.’*

(Social care practitioner)

Many practitioners, particularly the youngest, expressed reservations about telling older people how to live healthily:

*‘I do naturally have a great respect for older people, because they’ve been through it, they don’t want a younger person trying to tell them how to live their lives, when they’ve done it quite successfully for the last seventy years.’*

(Pharmacist)

Interviewees emphasised older people’s right to use alcohol and take risks. This perspective could deter intervention; and was particularly evident among domiciliary carers. Supporting older people to maintain their established lifestyle was at the heart of their role. Practitioners involved in alcohol intervention focused on ensuring older people’s decisions about drinking were informed:

*‘Ultimately, they’re adults … it is their responsibility …* [we are] *making sure that they’ve got that information so they can make their own informed choice.’*

(Nurse)

Most practitioners were acutely aware of the potential roles of alcohol in older people’s social lives and stress management, which contributed to their emotional wellbeing. Alcohol was central to many older people’s social activities, and a source of pleasure at a time in later life when social opportunities may have been limited, and stresses such as bereavement, loneliness, and boredom were common. Some practitioners described making allowances for their older care recipients based on such considerations, and acknowledged that they were less motivated to suggest limiting drinking:

*‘I often weigh up a risk–benefit and* [am] *probably guilty of turning a blind eye to the men who are going out drinking with their mates. There’s a balance … If getting pissed on a Friday is the price they pay to avoid total social isolation, I will roll with that.’*

(GP)

Drinking to cope with stresses was broadly perceived by practitioners to be a concern warranting intervention.

Practitioners with clear roles in screening and intervention, and appropriate training to equip them in this work (such as practice nurses and pharmacists), often possessed a belief that addressing alcohol use was part of their key care responsibilities. These practitioners were motivated to discuss alcohol use regardless of their reservations. Through experience, practitioners’ preconceptions about older people’s receptivity to alcohol-related discussion were challenged:

*‘Sometimes we worry about raising it because we assume patients are going to feel judged but doing it more often it’s much easier.’*

(GP)

An established rapport with older individuals, either through longer consultations (for example, medicine use reviews) or long-term practitioner–patient relationships (common in general practice), was felt to increase the acceptability of alcohol-related discussion:

*‘I’ve been seeing my* [older] *patients for thirty-five years. I’ve earned over the years enough credibility to say, “Look, let’s talk like a couple of grown-ups who know each other very well.” I’ll pretty much always get away with it. I find it very easy to raise questions about alcohol. An experienced GP, I would think, is one of the ideal people to raise it, because of that relationship that they have with patients.’*

(GP)

#### Processes and practicalities of addressing alcohol use

Practitioners described reminders to raise alcohol use with older people in their practice, including alcohol awareness campaigns and on-screen ‘pop-ups’ in general practice. They would raise concerns about alcohol use when faced with clear-cut symptoms. But there were other ways in which harmful drinking might present itself in older people — such as tremors or an unkempt home — that also prompted discussions about alcohol use:

*‘People presenting with certain symptoms and likely conditions, you would bring up alcohol. If someone was yellow, or if someone had abdominal symptoms, pain, diarrhoea — I’d probably bring up alcohol.’*

(GP)

Integrating alcohol-related discussion could be problematic in primary care, where practitioners were responsible for a range of care needs. Ensuring that older people understood their alcohol intake and associated health risks, and providing any necessary support, was perceived to be time consuming. Time constraints limited opportunity to discuss alcohol use when caring for older people, as multimorbidity was common with this group, and management of health conditions had to be prioritised:

*‘If we had enough time with older people, alcohol would be in it but you would be looking at all their conditions. If they’ve got multimorbidity, alcohol, yes, you would talk about, but sorting them out properly would probably be on your priority list as well. So time is a real constraint. You’ve got to be really careful what you do in ten minutes. If alcohol prevention had to be higher up, you definitely would need a longer consultation.’*

(GP)

For other practitioners, general health monitoring appointments created space for alcohol-related discussion. Dedicated time in ‘health checks’ for older adults allowed this potentially sensitive topic to be introduced as part of standard care, which tended to be accepted:

GP4: *‘They’re* [the patient] *probably expecting to be asked in those sorts of clinic situations where it’s a health check or something. Whereas I think with us* [GPs] *, they’re coming in with something that they don’t think is related to alcohol.’*

Nurse 2: *‘They might think, “Do I look like a drinker? I’ve only come for a sore on my finger.”’*

GP4: *‘I think it means we don’t* [raise alcohol use] *as much.’*

GP5: *‘If it’s normalised it’s good because it seems to be more acceptable if nursing staff are asking than medical staff because we ask it less.’* (GPFG)

Intervention options, or signposting opportunities for hazardous drinking, were considered important in ensuring that any needs for support were met. When these were not available, practitioners were more reluctant to raise alcohol use with older people:

*‘If you can intervene and say, “This is really important and I want you to go and see these people, make an appointment, and that phone number. Do you promise to do that?” that will increase the value of what you’re doing hugely. You have to have that pathway for people to follow.’*

(GP)

#### Professional remit and addressing older people’s alcohol use

Alcohol-related health promotion was a specific responsibility for a number of practitioner groups, and was prioritised in their interactions with older care recipients (see Supplementary Box S1). Everyone else cited barriers, such as time constraints and how receptive older people would be, as influences on the likelihood that they would discuss alcohol use.

Although older people’s support needs were often recognised by practitioners, they were wary of their remit and capability relative to other practitioners for identifying and/or intervening to address potentially risky alcohol use. These individuals tended to view elements of addressing older people’s alcohol use as others’ professional responsibility. Some practitioners expressed a lack of confidence about intervention, or understanding of low-risk alcohol use, especially with older people. This limited their ability to provide support:

*‘I’m not sure that I know enough about how good or bad alcohol is for you to be able to justify going any further with* [intervention] *apart from, “Did you know alcohol is linked to mouth cancer?” That’s as far as it goes really. I don’t go into any depth or any detail because I don’t have the knowledge.’*

(Dentist)

Professionals with less training in alcohol intervention, such as dentists, felt that there were others who were better equipped to meet older people’s support needs to make healthier decisions about alcohol use. Pharmacists and GPs demonstrated more extensive knowledge that informed their practice.

Practitioners working in social care for older people did not intervene directly and relied on referrals to health care. However, they described little success in prompting intervention this way as their concerns were often not shared:

DC1: *‘We don’t really have much luck with GPs. Normally, if you do a referral, you will get a visit, but GPs only really tend to be interested in illnesses.’*

DC2: *‘Acute illnesses, like an infection or a UTI.’*

DC1: *‘Where they can prescribe something. When it comes to other things, I think they tend to just pass it on.’* (Domiciliary care team)

Practitioners working in general practice acknowledged their role in managing older care recipients’ alcohol use. Organisation of services meant allied health professionals, such as nurses and healthcare assistants, were more likely to address older patients’ alcohol use through health screening and brief intervention initiatives for older people:

HCA2: *‘I think we* [healthcare assistants] *ask* [about alcohol use] *because it’s part of our sequence and it’s part of what we have to ask. It’s ingrained — every contact you’re saying, “Can we just ask you about your smoking, your drinking?”’*

GP4: ‘ *It’s not something that flags up though on our* [GPs’] *systems unless it’s become an official problem. It’s not all that obvious if we’re seeing them for something else unless we actually look for it.’* [GPFG]

## DISCUSSION

### Summary

To the authors’ knowledge, this is the first study to shine a spotlight on the facilitators and challenges affecting primary care practitioners’ work to support older people with decisions about their alcohol use. Findings provide a comprehensive and in-depth account of previously unexplored issues. They reflect the perspectives of a wide range of practitioners involved in addressing alcohol use as part of standard care for older people, including, and reaching beyond, GPs, who traditionally led in addressing alcohol use, and have been the focus of previous studies of factors affecting alcohol-related discussion in primary care.^28^ Practitioners’ perceived roles in addressing older people’s alcohol use differed between professions. Where alcohol-related discussion was not integral to their work, concerns about older people’s sensitivity to discussing the topic, fatalistic views about their ability to make lifestyle changes in old age, and competing priorities in addressing older people’s complex health needs in time-constrained care could all deter practitioners from addressing alcohol use with older adults. Practitioners with specific responsibility for alcohol screening and intervention in their care of older people had greater opportunity to provide support, with allocated time to broach the topic. They were often more confident, having had training for alcohol-related health risks and intervention. Practitioners were aware of the range of expertise in primary care involved in supporting older people, and the value of a multidisciplinary team to collectively provide any necessary support.

### Strengths and limitations

This in-depth qualitative study produced a rich understanding of factors affecting primary care practitioners’ work to support older people’s decisions about their alcohol use. Perspectives from a range of care professions enabled the identification of practitioner groups with capacity to support older people, and others who may need support to fulfil roles. Drawing on the COM-B model^27^ during analysis aided identification of factors likely to affect practitioners’ work to support older people’s decisions.

This study examined practice in primary care in Northern England, where alcohol use is a significant public health concern owing to high rates of alcohol-related harm relative to the rest of England.^3^^,^^29^ Opportunities and constraints to practitioners’ work with older people will vary across geographical areas, although some universal influences on practice have been identified.^14^

### Comparison with existing literature

Practitioners described particular challenges in addressing alcohol use with older people, including reservations about the positive contribution of alcohol to their lives in old age, how receptive older people are to intervention, their particular sensitivities about discussing alcohol use, and prioritising older people’s right to self-determine their lifestyle. Additional identified barriers, such as time constraints and insufficient training, are known to affect alcohol-related practice irrespective of patient age.^14^^,^^24^^,^^28^^,^^30^^–^^35^ However, the extensive healthcare needs of many older people, and particular challenges of addressing alcohol use with this age group, may mean these issues are accentuated in practice with older adults. Where such barriers have been identified in previous studies, it did not follow that older people’s needs for support with their alcohol use were met.^14^^,^^16^^,^^36^ In this study, barriers were overcome by roughly half of practitioners, ensuring the topic was broached and intervention provided. This may be because practitioners in UK primary care have a longer established role in addressing alcohol use than in previously studied care systems.^17^ Many practitioners had clear opportunities for alcohol-related discussion, particularly with older drinkers, as this was integrated within their standard care of older adults (for example, through alcohol screening and intervention during NHS Health Checks, Long Term Condition Reviews, and Medicine Use Reviews).^37^

Providing support for decisions about alcohol use was traditionally the role of GPs.^38^ The present findings indicate GPs often have capability, through skills and established relationships with older patients to support their decisions about alcohol use. However, workforce shortages, time pressures, and responsibilities for addressing multiple health concerns limit their capacity to provide support. Limited opportunity to address alcohol use with older people was the key barrier for GPs, rather than sensitivities around alcohol-related discussion, emphasised as a major demotivator to GPs’ work to address alcohol use in a previous study.^28^ In the absence of leading roles in alcohol screening programmes, GPs depended on visible signs to identify problematic drinking, which are linked to alcohol dependence, and may explain why hazardous alcohol use (usually without visible signs) is often undetected in older adults.^39^ There is increasing recognition of the roles of other practitioners in informing healthy lifestyle choices,^15^^,^^40^^,^^41^ including pharmacists and practice nurses, who may have protected time within appointments with older adults to discuss alcohol use. They are well equipped with the necessary skills and clear opportunity to support older people’s decisions.^41^ Other practitioner groups, such as dentists, had roles in addressing older people’s alcohol use, but their capability to fulfil this responsibility was limited. Practitioners with roles in addressing alcohol use were reliant on their professional expertise about risks of drinking among older care recipients; their level of knowledge was variable. Few practitioners conveyed awareness of the specific risks of drinking in old age; this is consistent with findings that health professionals are poor at identifying hazardous alcohol use among older people.^39^ Tools screening for specific risk factors for alcohol-related harm among older people, such as the Comorbidity Alcohol Risk Evaluation Tool (CARET), were not utilised by any practitioners, but they may be a useful guide for identifying potentially problematic use.^42^ Practitioner training courses about alcohol-related risks and intervention skills, such as those supplied by the Drink Wise, Age Well programme,^10^^.^^43^ may support practitioners in fulfilling their roles in supporting older people’s decisions.^27^

Although this study’s findings highlighted opportunities to support older people’s decisions about alcohol use in UK primary care, wider evidence suggests practitioners may not capitalise on these. Only one-third of older people screened for hazardous alcohol use during NHS Health Checks receive advice or feedback about their drinking.^44^ Failing to raise concerns about alcohol intake following health screenings can provide a false sense of assurance to older people that their alcohol intake is safe.^15^ Other barriers identified within this study, such as practitioners’ reservations about older people’s openness to alcohol-related discussion and making healthy lifestyle changes, may mean opportunities to motivate healthier decisions about alcohol use are not always taken. The present findings indicate that ageism in alcohol practice, demonstrated already within specialist services,^45^ extends to alcohol-related harm prevention initiatives. Practitioners articulated age-related preconceptions regarding older people’s sensitivities and receptivity, and reservations about roles of alcohol, which demotivated alcohol-related discussion with older people; this implies that explicit age discrimination was at play in their practice.

Practitioners were receptive to the potential positive contribution of alcohol to older people’s social and emotional wellbeing, in the context of reduced social networks and stress associated with retirement, bereavement, and other life transitions in old age. Some of these life circumstances are identified by older people as motivators for alcohol use in old age.^18^^,^^46^^,^^47^ However, such concerns can also prevent practitioners raising the subject of alcohol use. Reservations about how receptive older people are to intervention are contradicted by evidence that demonstrates older people’s engagement with, and ability to benefit from, support.^19^ Applying shared decision making may be particularly beneficial in addressing older people’s alcohol use, helping older people to explore how the positive roles of alcohol in their lives might be maintained, while limiting alcohol use to benefit their health and wellbeing.^15^^,^^48^ Practitioners’ concerns about older people’s social and emotional wellbeing might be addressed through social prescribing, a key component in UK disease prevention and loneliness initiatives.^17^^,^^49^ Signposting opportunities for further support were important to ensuring practitioners felt able to address alcohol use with older people. However, services to support people with problematic alcohol use can be inaccessible to older adults,^45^ and awareness of and referral to appropriate services can be inconsistent between primary care practitioners.^50^

### Implications for research and practice

This research demonstrates capacity in primary care to provide older people with support to make healthier decisions about alcohol use. Relevant intervention skills and knowledge of specific risks of drinking for older people, and clear opportunities to address alcohol use in the care of older adults, are essential. Practitioners with the time and relevant knowledge or skills to support older people’s decisions for alcohol use, such as practice nurses and pharmacists, are important assets who could have key roles in future initiatives in primary care to address alcohol-related harm among older people. All practitioners with roles in addressing older people’s alcohol use should be supported to overcome any challenges affecting their motivation, capability, and opportunities in providing support with appropriate training for their role. Preconceptions among practitioners about older people’s receptivity to intervention, which may demotivate work to address alcohol use with older people, must be challenged. Future research may examine how practitioners’ roles can be developed to provide older people with appropriate support for decisions about alcohol use, and could address challenges affecting practice.
